# FIP200 is Involved in Murine *Pseudomonas* Infection by Regulating HMGB1 Intracellular Translocation

**DOI:** 10.1159/000362954

**Published:** 2014-05-20

**Authors:** Yi Li, Chang-pei Gan, Shuang Zhang, Xi-kun Zhou, Xue-feng Li, Yu-quan Wei, Jin-liang Yang, Min Wu

**Affiliations:** a State Key Laboratory of Biotherapy, West China Hospital, West China Medical School, Sichuan University, Chengdu, China; b Department of Basic Sciences, University of North Dakota, Grand Forks, ND, USA

**Keywords:** FIP200, HMGB1 acetylation, Acute lung injury, Pulmonary infection

## Abstract

**Background:**

FIP200, a critical autophagy initiating protein, can participate in numerous cellular functions including cancer development; however, its functional role in *P. aeruginosa* infection of alveolar macrophages is unknown.

**Methods:**

To investigate the role of FIP200 in host defense, we transfected murine alveolar macrophage MH-S cells with FIP200 siRNA. Having confirmed that FIP200 knockdown inhibited PAO1-induced autophagosme formation, we sought to characterize the underlying signaling pathways by immunoblotting. Further, we used *fip200* KO mice to study the effects of *fip200* deficiency on HMGB1 translocation.

**Results:**

We showed that *Pseudomonas* PAO1 strain infection facilitated autophagosome formation, whereas knockdown of FIP200 inhibited autophagosome formation and HMGB1 expression in MH-S cells. Silencing FIP200 impaired the translocation of HMGB1 to cytosol of MH-S cells and almost abolished acetylation of HMGB1 during PAO1 infection. In contrast, FIP200 overexpression facilitated the cytosol translocation of HMGB1 from nuclei and increased acetylation of HMGB1 in PAO1-infected MH-S cells. Importantly, expression and acetylation of HMGB1 were also significantly down-regulated in *fip200* KO mice following PAO1 infection.

**Conclusions:**

Collectively, these findings elucidate that FIP200 may regulate expression and translocation of HMGB1 during PAO1 infection, which may indicate novel therapeutic targets to control pulmonary infection.

## Introduction

*Pseudomonas aeruginosa* is commonly isolated from patients with hospital-acquired infection, and causes serious consequence in cystic fibrosis (CF) [1]. Treatments of this infection are usually difficult due to the impairment of multiple components in host immunity and fast development of antibiotic resistance, but the pathogenesis mechanism with this pathogen is incompletely understood [2, 3]. Since alveolar macrophages (AM) are the sentinel of the initiation and integration of immune responses to microbial infection, we aimed to understand the molecular pathogenesis involved in AM defense against *P. aeruginosa* [4].

High mobility group box 1 (HMGB1) is a highly conserved, ubiquitous protein that is expressed in nearly all cell types. Not only can HMGB1 bind to double-stranded DNA and interact with other DNA-binding proteins to facilitate chromatin binding, but also function as a nuclear factor to enhance transcription in response to infection, inflammation, and tissue injury [5, 6]. HMGB1 is a potential therapeutic target of local and systemic inflammatory diseases including acute lung injury, epithelial barrier dysfunction, and arthritis [7]. Extracellular HMGB1 released from inflammatory cells or necrotic cells can stimulate macrophages to secrete cytokines that can further amplify inflammatory responses [8]. In inflammatory diseases, such as sepsis, HMGB1 is translocated from the nucleus to the cytoplasm and actively secreted into the extracellular environment, where it interacts with several surface molecules, including Receptor for Advanced Glycation End-products (RAGE) and Toll-like Receptor 4 (TLR4) [9, 10]. Increased HMGB1 expression has been linked to infection progression by interfering with several signaling pathways, especially the autophagy pathway [11]. Huang et al. have reported that HMGB1-mediated autophagy contributed to chemotherapy in osteosarcoma by controlling the formation of Beclin1-Phosphatidylinositol 3-kinase Class 3 (PI3K3) complex [12]. As an upstream signal, Focal adhesion kinase family interacting protein of 200 kDa (FIP200) is required for the interaction between HMGB1 and Beclin1, which then promotes Beclin1-PI3KC3 complex formation during autophagy [13].

Autophagy is essential for various cellular processes and associated with many human diseases, such as colon cancer, hepatitis B virus-associated hepatocellular carcinoma, diabetes, pulmonary infection, etc. [14–18]. FIP200, also known as RB1CC1 or RB1-inducible coiled-coil, is a component of the ULK1-Atg13-FIP200 complex, which is an essential autophagy initiator in mammalian cells [19]. Previous studies demonstrate that FIP200 is required for autophagy flux induced by infection in macrophages [20].

The role of FIP200 in the activation of macrophages during *P. aeruginosa* pulmonary infection remains unclear. Identifying effects of FIP200 on the production of HMGB1 by macrophages may help understand the molecular pathogenesis of *P. aeruginosa* infection. This study is designed to analyze the effects of FIP200 on HMGB1 translocation in macrophages during *P. aeruginosa* infection.

## Materials and Methods

### Reagents

*P. aeruginosa* strain PAO1 wild-type (WT) was a gift from Stephen Lory (Harvard Medical School, Boston, MA). GFP-PAO1 strain was obtained from Gerald Pier (Channing Laboratory, Harvard Medical School) [21]. The myc-FIP200 plasmid was a gift from DoHyung Kim (University of Minnesota, Minneapolis). The tandem RFP-GFP-LC3 plasmid was created and kindly provided by Tamotsu Yoshimori of Osaka University, Japan [22].

### Cell culture

MH-S, a mouse macrophage-like cell line, was obtained from American Type Culture Collection (ATCC, Rockville, MD) and maintained following the supplier’s instructions [23]. These cells were grown in 1640 containing 10% FBS, 100 units/ml penicillin, and 100 μg/ml streptomycin in a 95% air, 5% CO_2_ humidified atmosphere at 37 °C [24].

### Establishment of a murine model with P. aeruginosa infection

Cre-mediated-FIP200 conditional knockout (KO) mice were obtained from Dr. Junlin Guan at University of Michigan Medical School as described previously [25–27]. The conditional KO mice were generated by Cre-loxP approach, which mediated FIP200 deletion in endothelial cells [25–27]. Mice genotyping for FIP200 were performed by polymerase chain reaction analysis of tail DNA, essentially as described previously [26]. Six to eight week-old wild-type (WT) mice (C57BL/6J) were obtained from The Jackson Laboratory (Bar Harbor, ME) [28]. Mice were housed and bred in the animal facility at the University of North Dakota, and the animal experiments were performed in accordance with the institutional animal care and use committee guidelines (IACUC, approval number 1204-4). Bacteria were grown in Luria–Bertani (LB) broth overnight at 37 °C with shaking. The bacteria were pelleted by centrifugation at 8000 *g* next day. To make sure that the bacteria grow until the mid-logarithmic phase, they were resuspended in 10 ml of fresh LB broth [29]. We anesthetized mice with 45 mg/kg ketamine [30] and intranasally instilled to 1×10^7^ (PAO1) colony-forming units (CFU) of *P. aeruginosa*, and mice were killed at 48 h following infection [31]. After bronchoalveolar lavage (BAL), the trachea and lung were excised for homogenization or fixed in 10% formalin.

### Infection experiments

Bacteria were grown in LB broth overnight at 37 °C with shaking and pelleted by centrifugation at 8000 *g* next day. The optical density (OD) at 600 nm was measured, and the density was adjusted to 0.25 OD (0.1 OD=1×10^8^ cells/ml). MH-S cells were washed once by PBS after overnight culture in serum-containing medium and changed to serum-free and antibiotic-free medium immediately before infection. Then cells were infected by PAO1 at an MOI (Multiplicity of Infection) of 10:1 for 3 h. The CFU assay was performed after treating the infected cells with 100 μg/ml of polymyxin B [31, 32].

### RNA isolation and RT-PCR

Total RNA was isolated from lung tissue using TRIzol reagent (Invitrogen, Carlsbad, CA) following the manufacturer’s instructions. The concentration of RNA was determined and cDNA was generated using total RNA with the Reverse Transcriptase kit (Invitrogen). For amplification of the desired cDNA, specific primers were listed in Table 1. RT-PCR products were visualized on 1% agarose gels containing ethidium bromide following electrophoresis [33, 34].

### Cell transfection and confocal microscopy

MH-S cells were respectively transfected with RFP-LC3 and RFP-GFP-LC3 plasmids, using LipofectAmine 2000 reagent (Invitrogen) in serum-free RPMI 1640 medium (Thermofisher Scientific, San Jose, CA) following the manufacturer’s instructions. Transfected MH-S cells were also starved (6 h) or treated with the autophagy inhibitor 3-MA (3 mM, 3 h) as a positive and negative control, respectively. For immunostaining, MH-S cells were grown in glass-bottomed dishes (MatTek, Ashland, MA) and fixed in 3.7% paraformaldehyde, permeabilized with 0.2% Triton X-100 in PBS and incubated with blocking buffer for 30 min [35]. Then the dishes were incubated with an HMGB1 antibody (Santa Cruz Biotechnology, Santa Cruz, CA) at 1/500 dilution in blocking buffer overnight and washed three times with wash buffer. Images were captured after incubation with an appropriate secondary antibody containing FITC using an LSM 510 Meta confocal microscope (Carl Zeiss MicroImaging, Thornwood, NY), and processed using the software provided by the manufacturer [22]. The confocal microscopy images were then used to semi-quantitatively measure the percentage of cells with significant LC3 punctation staining (100 cells/sample). The threshold for positive expression was set to 10 visible LC3 puncta.

### MTT assay

MH-S cells were seeded at a density of 5×10^3^ per well in 96-well plates (Costar Corning, Rochester, NY). After incubation overnight, cells were transfected with FIP200 siRNA and control siRNA (Santa Cruz Biotechnology), respectively. Then cells were infected by PAO1 at an MOI of 10:1 for 3 h. 5 wells were included in each group. After 4 h incubation with 3-(4,5-dimethylthiazol-2-yl)-2,5-dimethyltetrazolium bromide dye at a final concentration of 5 μg/ml per well, the absorbance at 570 nm was measured with SpectraMax M5 (Molecular Devices), using wells without cells as blanks.

### Isolation of nuclear extracts

Nuclear extracts were isolated following the instruction of NE-PER Nuclear And Cytoplasmic Extraction Reagents Kit (Pierce-Thermofisher Scientific). Cells were washed by suspending the cell pellet with phosphate-buffered saline. We added the CER I buffer and CER II buffer to swell the cells on ice for 10 min and then vortexed for 10 s. Subsequently, samples were centrifuged for 10 s and the supernatant fraction was discarded. Pellets were resuspended in NER buffer and incubated on ice for 40 min for high-salt extraction. Cellular debris was removed by centrifugation for 10 min at 16,000 *g* and the supernatant fraction (containing DNA-binding proteins) was stored at −80 °C until use. Protein concentrations were determined by the BCA Protein Assay Kit (Bio-Rad, Hercules, CA) [36].

### Western blot

The samples from cells or tissues were lysed and quantified. Twenty micrograms of protein from murine lung tissue or MH-S cells were mixed with an equal volume of 2×SDS sample buffer, boiled for 5 min and then separated by 10% SDS-polyacrylamide gel electrophoresis (PAGE). After electrophoresis, proteins were transferred to nitrocellulose membranes (Santa Cruz Biotechnology). Membranes were incubated with primary antibodies against FIP200, LC3, Beclin1, RAGE, ULK1, Histone, TLR4 and GAPDH (Santa Cruz Biotechnology). The antibodies against Atg13 and HMGB1 were from Cell Signaling Technology (Danvers, MA). Western blotting rabbit polyclonal antibodies, mouse polyclonal antibodies and goat polyclonal antibodies were obtained from Santa Cruz Biotechnology. Signals were visualized using an enhanced chemiluminescence detection kit (Santa Cruz Biotechnology). The protein signal was quantified by scanning densitometry using Quantity one imaging analysis (Bio-Rad).

### Immunoblotting and immunoprecipitation

To obtain whole-cell lysates, MH-S cells or murine lung homogenates were homogenized in lysis buffer containing phosphatase inhibitor (1:1000) and Protease inhibitors (1:50, Roche, Indianapolis, IN). The samples were centrifuged at 12,000 rpm for 10 min at 4 °C. Protein concentrations were determined by the BCA Protein Assay Kit (Bio-Rad) and then stored at −80 °C for immunoblotting analysis [37].

Whole-cell lysates were mixed with anti-FIP200 antibody (Cell Signaling Technology, Beverly, MA) or anti-HMGB1 antibody (Santa Cruz Biotechnology), respectively, which were coupled to agarose beads (Invitrogen). Nuclear Extracts were mixed with anti-acetylation on epsilon-amine groups of lysine residues antibody (Cell Signaling Technology) or anti-HMGB1 antibody (Santa Cruz Biotechnology) coupled with agarose beads. Immunoprecipitates were separated by SDS-PAGE and transferred to nitrocellulose transfer membranes (Santa Cruz Biotechnology). Signals were visualized using an ECL kit (Santa Cruz Biotechnology). The protein signal was quantified by scanning densitometry using Quantity one imaging analysis (Bio-Rad).

### Statistical analysis

Statistical analysis was performed with the SPSS software system (SPSS for Windows, version 13.0; SPSS Inc., Chicago, IL). Parametric data were statistically analyzed by one-way ANOVA followed by post hoc tests. Differences in Non-parametric data were evaluated by the Mann-Whitney *U* test. Data were expressed as means ± SD. A significant difference was defined as *p* < 0.05 [38].

## Results

### FIP200 knockdown inhibited PAO1-induced autophagosome formation and impaired phagocytosis function of MH-S cells

**Fig. 1:**
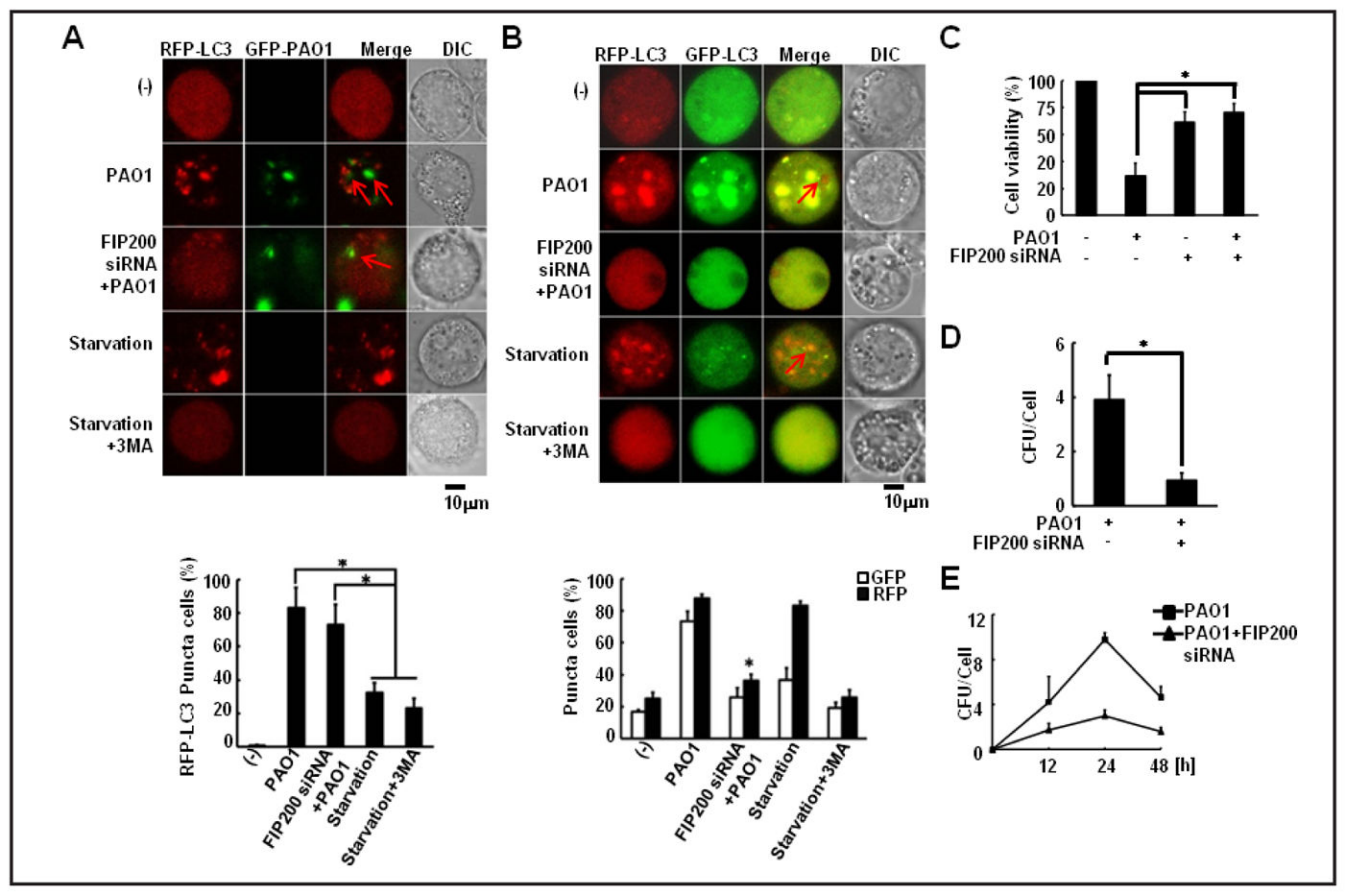
FIP200 knockdown inhibited PAO1-induced autophagosome formation and impaired phagocytosis function of MH-S cells. A. MH-S cells were transfected with an RFP-LC3 plasmid and then infected for 3 h with GFP-PAO1 at an MOI of 10:1 induced significant LC3 punctation in MH-S cells. Transfected MH-S cells were also serum starved (6 h) or treated with the autophagy inhibitor 3-MA (3 mM, 3 h) as a positive and negative control, respectively. Before infection, the cells were transfected with FIP200 siRNA. B. MH-S cells were transfected with an RFP-GFP-LC3 plasmid and infected with PAO1 at an MOI of 10:1. Transfected MH-S cells were also serum starved (6 h) or treated with the autophagy inhibitor 3-MA (3 mM, 3 h). Before infection, the cells were transfected with FIP200 siRNA. C. FIP200 silencing improved MH-S cell survival upon PAO1 infection. Cells were infected by PAO1 at an MOI of 10:1 for 3 h and MTT assay was performed to determine host cell (MH-S) viability. Before infection, the cells were transfected with FIP200 siRNA. D. MH-S cells were infected by PAO1 at an MOI of 10:1 for 3 h and phagocytosis assay was performed. Before infection, the cells were transfected with FIP200 siRNA. E. MH-S cells were tested by phagocytosis assay in a time-dependent manner (0, 12, 24 and 48 h) upon PAO1 infection at an MOI of 10:1. Before infection, the cells were transfected with FIP200 siRNA. The data represent means±SD of 3 independent experiments (one-way ANOVA followed by post hoc tests). Statistically significant differences are indicated by * *p*<0.05.

To investigate the role of FIP200 in alveolar macrophage defense against *P. aeruginosa* infection, we transfected murine MH-S cells with an RFP-LC3 plasmid and observed that PAO1 infection with an MOI of 10:1 induced significant LC3 punctation (83.33±12.05%). However, FIP200 knockdown by siRNA resulted in a substantial downregulation of autophagy following PAO1 infection with a decrease (32.33±6.11%) of RFP-LC3 puncta (Fig. 1A). We next utilized a tandem RFP–GFP–LC3 construct to validate the observation and found that FIP200 knockdown affected autophagosome formation. The RFP–GFP–LC3 plasmid was designed to differentiate two major autophagic vesicles, the autophagosome and the autolysosome, thus excluding the likelihood of simple lysosome degradation blockade. When an autophagosome fuses with a lysosome, the GFP moiety degrades from the tandem protein, but RFP–LC3 maintains the red puncta to track autolysosomes [22]. After successful transfection with the tandem construct, FIP200 siRNA transfection significantly reduced red puncta (36.67±3.51%) in MH-S cells compared to control siRNA transfection (88.33±1.53%) (Fig. 1B). PAO1 infection significantly decreased MH-S cell survival; however, FIP200 silencing improved MH-S cell survival after PAO1 invasion (Fig. 1C). FIP200 siRNA-transfected MH-S cells significantly decreased CFU after PAO1 infection (Fig. 1D). Finally, we observed that FIP200 knockdown significantly decreased CFU upon infection in a time-dependent manner (Fig. 1E). Overall, our results suggest that FIP200 may be required for PAO1-induced autophagosome formation and phagocytosis function in macrophages.

### FIP200 knockdown decreased the expression of HMGB1 during PAO1 infection

**Fig. 2:**
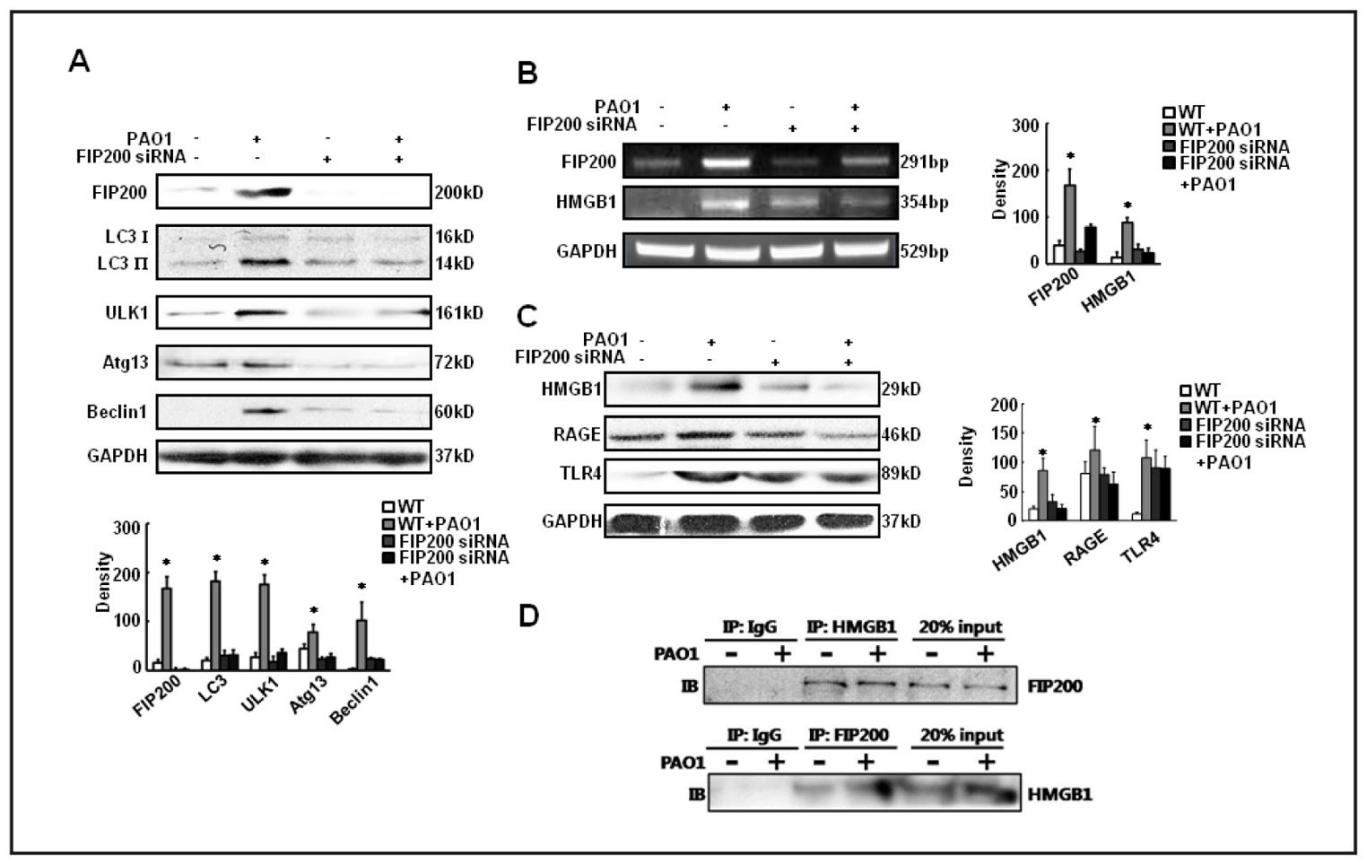
FIP200 knockdown decreased the expression of HMGB1 during PAO1 infection. MH-S cells were transfected with FIP200 siRNA or control siRNA and then infected with PAO1 at an MOI of 10:1 for 3 h. A. FIP200 knockdown decreased the expressions of LC3, Atg13, ULK1 and Beclin1 during PAO1 infection in MH-S cells. B. FIP200 silencing in MH-S cells significantly reduced mRNA levels of FIP200 and HMGB1 after PAO1 infection. C. FIP200 siRNA-transfection of MH-S cells significantly decreased expression in HMGB1, RAGE and TLR4 by western blotting. D. An interaction between FIP200 and HMGB1 identified by reciprocal coimmunoprecipitation. For western blotting we used HMGB1 antibody from Cell Signaling Technology (Danvers, MA). For immunoprecipitation we used anti-HMGB1 antibody from Santa Cruz Biotechnology. The data represent means±SD of 3 independent experiments (one-way ANOVA followed by post hoc tests). Statistically significant differences are indicated by * *p*<0.05.

Having revealed that FIP200 knockdown inhibited PAO1-induced autophagosme formation, we sought to characterize the underlying signaling pathways. We examined the involvement of several canonical autophagic proteins related to FIP200, such as LC3, Atg13, ULK1 and Beclin1. PAO1 infection up-regulated the expression of LC3, Atg13, ULK1 and Beclin1; however, FIP200 knockdown decreased the expressions of these proteins (Fig. 2A). We further analyzed the impact of FIP200 knockdown on HMGB1 expression. As shown in Fig. 2B, FIP200 silencing in MH-S cells significantly reduced mRNA level of HMGB1 after PAO1 infection (Fig. 2B). We also observed that FIP200 siRNA silencing in MH-S cells significantly decreased protein expression of HMGB1, RAGE and TLR4 following infection by western blotting (Fig. 2C). Thus we hypothesized that FIP200 may influence HMGB1 through molecular interaction. As expected, an interaction between FIP200 and HMGB1 occurred as identified by reciprocal coimmunoprecipitation (Co-IP) (Fig. 2D).

### FIP200 knockdown impaired HMGB1 translocation during infection

**Fig. 3:**
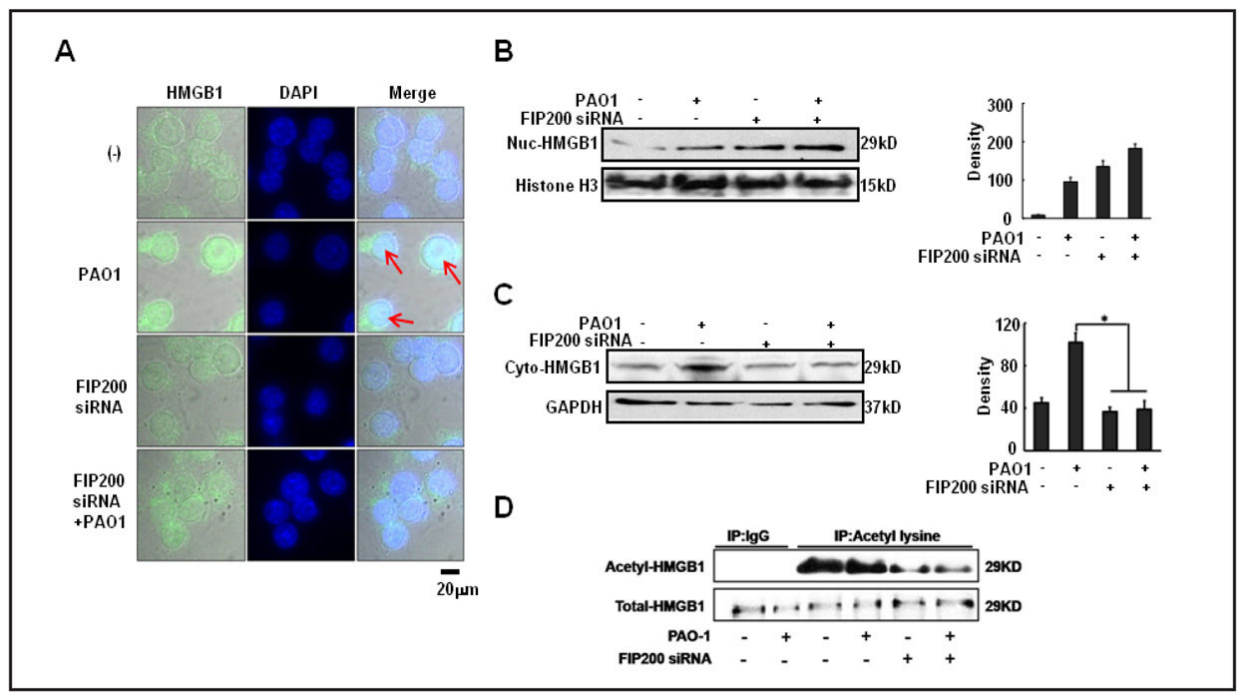
FIP200 knockdown impaired HMGB1 cytosolic translocation from nuclei upon infection. MH-S cells were transfected with FIP200 siRNA or control siRNA and then infected with PAO1 at an MOI of 10:1 for 3 h. A. FIP200 knockdown in MH-S cells inhibited HMGB1 translocation to cytosol upon PAO1 infection. B. Nuclear HMGB1 was increased in PAO1-infected MH-S cells upon FIP200 siRNA transfection. C. Cytosolic HMGB1 was decreased by FIP200 siRNA transfection post PAO1 infection. D. FIP200 siRNA transfection inhibited the acetylation of HMGB1. For western blotting we used an HMGB1 antibody from Cell Signaling Technology. For immunoprecipitation we used anti-HMGB1 antibody from Santa Cruz Biotechnology. The data represent means±SD of 3 independent experiments (one-way ANOVA followed by post hoc tests). Statistically significant differences are indicated by * *p*<0.05.

We next examined the functional role of FIP200 on HMGB1 translocation and also found that FIP200 knockdown in MH-S cells inhibited HMGB1 translocation from nuclei to cytoplasm upon PAO1 infection using immunocytochemistry staining (Fig. 3A). To mechanistically elucidate translocation of HMGB1 during PAO1 infection, we fractioned nuclear compartment from cytoplasm using a commercial kit (NE-PER Nuclear And Cytoplasmic Extraction Reagents). We observed that nuclear HMGB1 was increased by 86.67±12.42% in PAO1-infected MH-S cells after FIP200 siRNA transfection, whereas cytosolic HMGB1 was decreased by 63.00±10.44% following the same treatment (Fig. 3B and C). Importantly, FIP200 silencing impaired the acetylation of the epsilon-amine groups on lysine residues in HMGB1 (Fig. 3D). These data indicate that FIP200 may regulate HMGB1 translocation by molecular interaction.

### Overexpressing FIP200 facilitated cytosol translocation of HMGB1 during infection

**Fig. 4:**
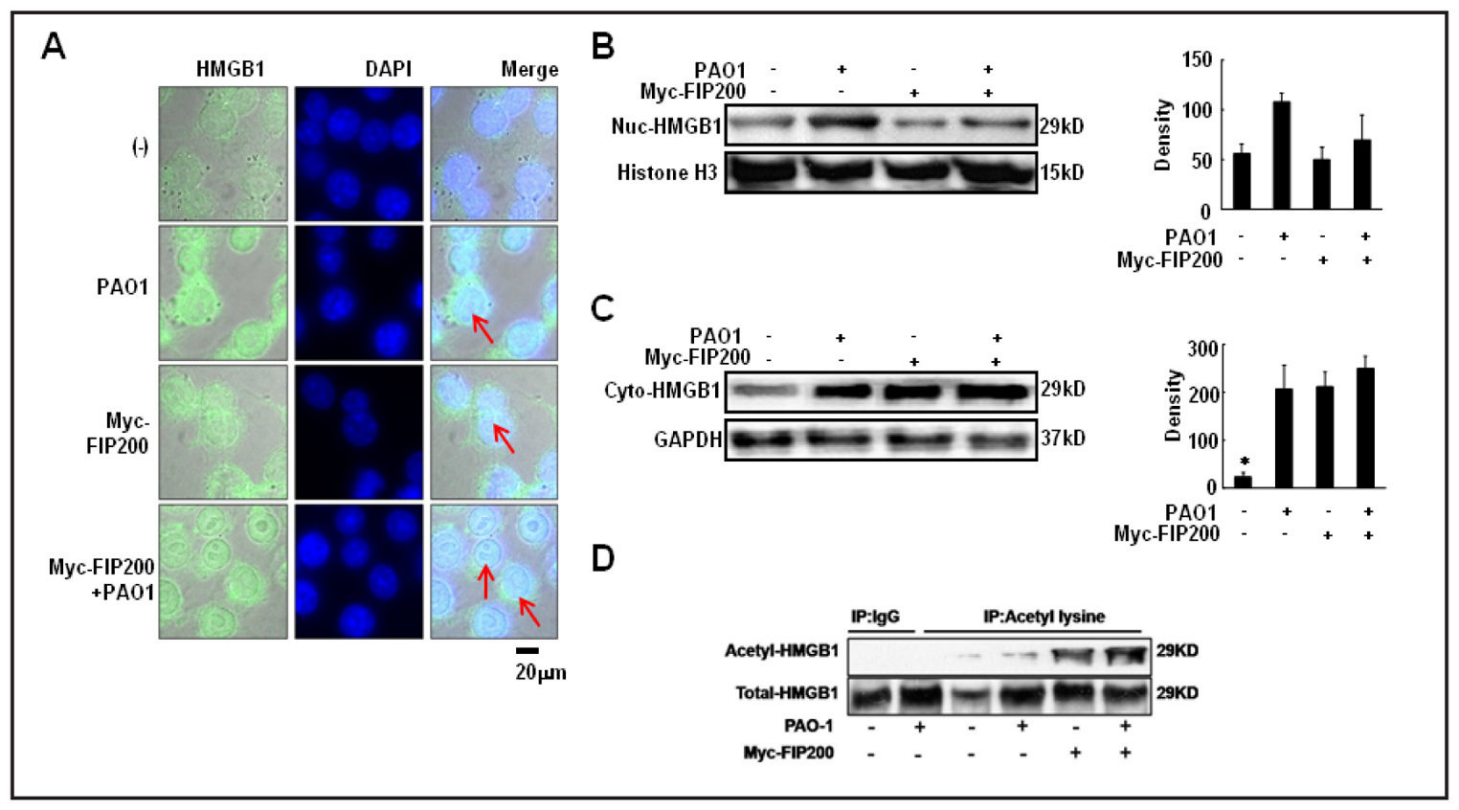
FIP200 overexpression facilitated HMGB1 translocation under infection. MH-S cells were transfected with a myc-FIP200 plasmid and then infected with PAO1 at an MOI of 10:1 for 3 h. A. myc-FIP200 plasmid transfection in MH-S cells facilitated HMGB1 translocation after PAO1 infection. B. Nuclear HMGB1 was decreased in MH-S cells upon myc-FIP200 plasmid transfection and by PAO1 infection. C. Cytosolic HMGB1 in MH-S cells was increased by myc-FIP200 plasmid transfection and by PAO1 infection. D. myc-FIP200 plasmid transfection induced the acetylation of HMGB1. For western blotting we used an HMGB1 antibody from Cell Signaling Technology. For immunoprecipitation we used an anti-HMGB1 antibody from Santa Cruz Biotechnology. The data represent means±SD of 3 independent experiments (one-way ANOVA followed by post hoc tests). Statistically significant differences are indicated by * *p*<0.05.

To elucidate the functional role of FIP200 on HMGB1 translocation, we transfected MH-S cells with a myc-FIP200 plasmid and observed that FIP200 overexpression facilitated the cytosol translocation of HMGB1 from nuclei upon PAO1 infection (Fig. 4A). Further, we detected that nuclear HMGB1 was decreased to 71.02±25.14% in PAO1-infected MH-S cells after myc-FIP200 plasmid transfection, whereas cytosolic HMGB1 was increased to 250.11±24.95% (Fig. 4B and C). Importantly, FIP200 overexpression increased the acetylation of the epsilon-amine groups on lysine residues in HMGB1 (Fig. 4D). These data indicate that FIP200 may positively regulate HMGB1 translocation by molecular interaction and acetylation of particular amino acids.

### fip200 deficiency was responsible for HMGB1 translocation in vivo

**Fig. 5:**
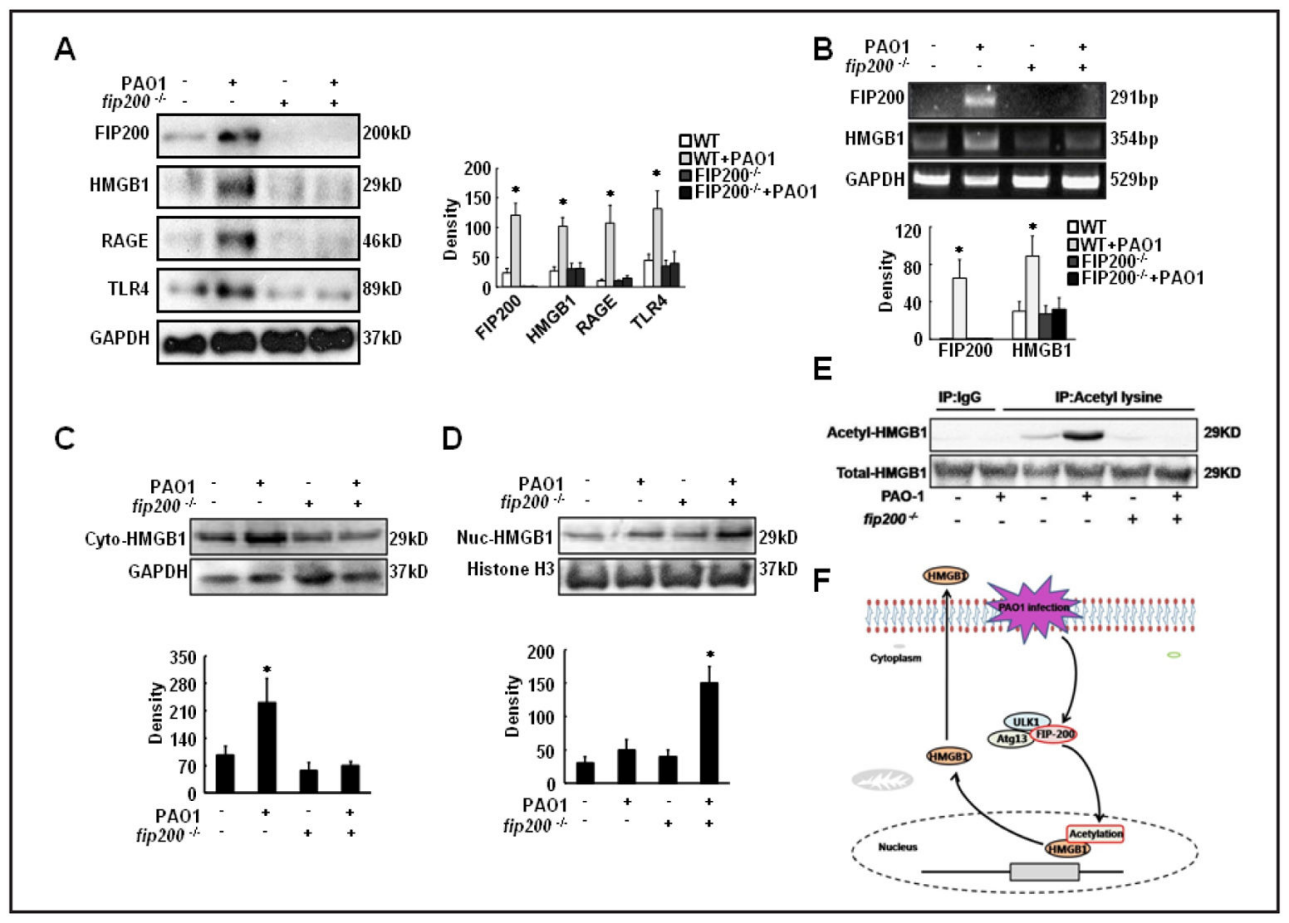
fip200 deficiency impaired HMGB1 translocation in vivo *fip200* deficient mice and WT mice (n=5) were intranasally infected with 1×10^7^ CFU/mice PAO1 for 48 h. A. *fip200* deficiency significantly reduced the expression of FIP200, HMGB1, RAGE and TLR4 in lung tissue following PAO1 infection. B. *fip200* deficiency significantly reduced mRNA level of HMGB1 in lung tissue following PAO1 infection. C. Cytosolic HMGB1 was decreased by *fip200* deficiency post PAO1 infection. D. Nuclear HMGB1 was increased in PAO1-infected MH-S cells upon *fip200* deficiency. E. *fip200* deficiency inhibited the acetylation of HMGB1. For western blotting we used an HMGB1 antibody from Cell Signaling Technology. For immunoprecipitation we used anti-HMGB1 antibody from Santa Cruz Biotechnology. The data represent means±SD of 3 independent experiments (one-way ANOVA followed by post hoc tests). Statistically significant differences are indicated by * *p*<0.05. F. Schematic illustration of FIP200-regulated HMGB1 translocation during *P. aeruginosa* infection.

To determine whether the response of HMGB1 pathway can occur *in vivo*, we used *fip200* KO mice to study the effects of *fip200* deficiency on HMGB1 translocation. As shown in Fig. 5A, *fip200* deficiency significantly reduced the expressions of HMGB1, RAGE and TLR4 in lung tissue after PAO1 infection. In addition, *fip200* deficiency significantly decreased mRNA level of HMGB1 upon PAO1 infection (Fig. 5B). Furthermore, cytosolic HMGB1 was decreased by 70.10±10.21%, whereas nuclear HMGB1 was increased by 150.11±25.03% in PAO1-infected *fip200* deficient mice (Fig. 5C and D). These data completely recapitulated the observation from cell models (Fig. 3 and 4). Furthermore, *fip200* deficiency impaired the acetylation of the epsilon-amine groups of lysine residues in HMGB1 (Fig. 5E), which is also consistent with the data obtained from cell culture (Fig. 3 and 4). Altogether, our data strongly suggest that FIP200 plays a similar function in Pseudomonal infection in mice as seen in cultured cells by promoting HMGB1 activation and cytosolic translocation, and thus depleting FIP200 inhibits HMGB1 cytosolic translocation.

## Discussion

In the current study, we demonstrated that FIP200 regulated HMGB1 translocation during *P. aeruginosa* infection. HMGB1 localized in the nucleus in resting cells, whereas PAO1 infection decreased its nuclear levels but increased cytosolic levels. Consistent with this result, HMGB1 is previously reported to be released by exciting innate immunity with exogenous pathogen-derived molecules [39]. Once released, HMGB1 can bind to cell-surface receptors, such as RAGE and TLR4, and elicit various cellular responses including chemotactic cell penetration and proinflammatory cytokine production [40, 41]. Tang et al. reported that autophagy stimuli could induce HMGB1 cytosolic translocation from the nucleus, indicating that autophagy was required to enhance the cytosolic translocation of HMGB1 [42]. Our findings showed that FIP200 silencing could suppress the expression of HMGB1, RAGE, K-Ras and TLR4 during PAO-1 infection. To further investigate the effect of FIP200 on the expression of HMGB1 in MH-S cells, we performed a Co-IP assay and found an interaction between FIP200 and HMGB1 following PAO1 infection. Thus, our data indicate that FIP200 may regulate HMGB1 translocation to cytosol following PAO1 infection via direct molecular interaction with HMGB1.

Acetylation of HMGB1 induced by lipopolysaccharide and hydrogen peroxide triggers molecular translocation within the cell [43]. When HMGB1 is not acetylated, it stays in the nucleus; however, acetylation of lysine residues causes HMGB1 to translocate into the cytosol. Here, we showed that PAO1 infection induced acetylation of the epsilon-amine groups of lysine residues in HMGB1 and nuclear translocation of HMGB1. Importantly, FIP200 silencing significantly reduced the acetylation of HMGB1 and inhibited HMGB1 translocation in MH-S cells upon PAO1 infection. FIP200 knockdown increased the level of nuclear HMGB1 in PAO1-infected MH-S cells, but decreased the level of cytosolic HMGB1. Furthermore, FIP200 overexpression facilitated cytosolic translocation of HMGB1 from nuclei and increased the acetylation of HMGB1 in PAO1 infected MH-S cells. To assess the physiological relevance, we examined the FIP200 functional role in bacterial infection in animals and obtained similar results in *fip200* deficient mice. Our study also suggests that FIP200 functioning in response to pathogen recognition facilitates HMGB1 acetylation, leading to translocation of HMGB1. Post-translational modification of HMGB1 including acetylation at the site of lysines 2 and 11 in cells modifies the binding of HMGB1 to DNA and its extranuclear localization [44]. Further research may identify the exact acetylation sites of HMGB1 upon FIP200 regulation.

FIP200, which regulates diverse cellular functions through its interaction with multiple other proteins, is also required for autophagy flux [45, 46]. However, the role of FIP200 in *P. aeruginosa* infection remains undefined. Here, we demonstrated that FIP200 could affect extracellular bacterial clearance by facilitating autophagy flux, as FIP200 knockdown significantly decreased bacterial burdens of murine alveolar macrophage MH-S cells. Despite focusing on alveolar macrophages, alveolar epithelial cells could also serve as an inflammatory and immune responder and play important roles in tissue injury [47]. Thus, further studies can also address the mechanisms that are responsible for FIP200 in alveolar epithelial cells upon *P. aeruginosa* infection.

In contrast to the intense interest in autophagy’s roles in cell viability, tumor, and inflammatory disease, little is known about how FIP200 regulates cellular functions in infectious disease. Our results showed that FIP200 siRNA transfection reduced the expression of HMGB1, RAGE and K-Ras in PAO1-infected MH-S cells. *fip200* deficiency decreased levels of HMGB1, RAGE and K-Ras in PAO1-infected mice. Mechanistically, we identified an interaction between FIP200 and HMGB1. Moreover, *fip200* deficiency impaired acetylation of HMGB1. Collectively, this study reveals that FIP200 upon PAO1 pulmonary infection down-regulates HMGB1 expression and its cytosolic translocation. Our findings suggest that FIP200 may play a regulatory role in alveolar macrophage-mediated host defense against *P. aeruginosa* infection, and serve as a target for therapy of Gram-negative bacterial infection.

## Figures and Tables

**Table 1:** Sequences of gene-specific primers used for PCR amplification

mRNA	Primer sequence
GAPDH	R 5′-TGCCTGCTTCACCACCTTCT -3′
	F 5- AGGCCGGTGCTGAGTATGTC -3
HMGB1	R 5′-CTGCTTGT-CATCTGCAGCAG -3′
	F 5′-GGAGGAGCATAAGAAGAAGC -3′
RAGE	R 5′-TGTGTGGCCAC-CCATTCCAG -3′
	F 5′-GCCCTCCAGTACTACTCTCG -3′
FIP200	R 5′-CGATCTGCAGCCATGCATTC -3′
	F 5′-GCCAATATCAAGCAAGCGCA -3′
